# Ovarian follicular response to oestrous synchronisation and induction of ovulation in Norwegian Red cattle

**DOI:** 10.1186/s13028-020-00514-6

**Published:** 2020-03-12

**Authors:** Halldor Felde Berg, Bjørg Heringstad, Anne Hege Alm-Kristiansen, Vilde Granne Kvale, Knut Ingolf Dragset, Andres Waldmann, Erik Ropstad, Elisabeth Kommisrud

**Affiliations:** 1 Department of Production Animal Clinical Sciences, Faculty of Veterinary Medicine, Norwegian University of Life Sciences, Ullevålsveien 72, 0454 Oslo, Norway; 2 Department of Animal and Aquacultural Sciences, Faculty of Biosciences, Norwegian University of Life Sciences, Arboretveien 6, 1430 Ås, Norway; 3 SpermVital AS, Holsetgata 22, 2317 Hamar, Norway; 4 Department of Biotechnology, Faculty of Applied Ecology, Agriculture and Biotechnology, Inland Norway University of Applied Sciences, Postbox 400, 2418 Elverum, Norway; 5 Geno Breeding and AI Association, Storhamargata 44, 2317 Hamar, Norway; 6 Institute of Veterinary Medicine and Animal Sciences, Estonian University of Life Sciences, Friedrich Reinhold Kreutzwaldi 62, 51006 Tartu, Estonia

**Keywords:** Norwegian Red, Oestrous synchronisation, Ovulation, Ultrasonography

## Abstract

**Background:**

Oestrous synchronisation of cattle has been widely applied to accomplish simultaneous ovulation in animals and facilitate timed artificial insemination. The main aim of this study was to investigate the ovarian follicular growth and ovulatory response to oestrus and ovulation synchronisation in Norwegian Red heifers and cows. Oestrous cycles in 34 heifers and 10 cows from 4 herds were synchronised with two PGF_2α_ analogue treatments 11 days apart, followed by GnRH analogue treatment for induction of ovulation. Thereafter, the ovaries were examined by ultrasonography at 3 h intervals until ovulation.

**Results:**

The luteolytic effect of the PGF_2α_ analogue was verified in 9 of 10 cows by progesterone contents in milk. Maximum physical activity of the cows occurred on average 69 h after PGF_2α_ analogue treatment. An ovulatory response was recorded in 95.5% (42/44) of the animals. A significant difference in follicle size at ovulation was found between 2 of the herds. Animals with medium sized and large follicles and heifers aged > 16 months ovulated earlier than other animals.

**Conclusions:**

The applied sequence of treatments in the study was shown to be effective in synchronizing and inducing ovulation within a relatively narrow time interval in the Norwegian Red heifers and cows, consistent with findings in other cattle breeds.

## Background

Synchronisation protocols are commonly applied in cattle to achieve simultaneous ovulation, allowing animals to be inseminated at a pre-set time, i.e. timed artificial insemination (AI). A variety of protocols have been developed for the reproduction management of cattle, especially in large herds, using different combinations of treatments with progesterone (P4), prostaglandin F_2α_ (PGF_2α_) and gonadotropin-releasing hormone (GnRH) to control the oestrous cycle and ovulation [1, 2]. Pre-synchronisation of follicular waves has been attempted using two-dose PGF_2α_ protocols and administering GnRH between PGF_2α_ treatments [3]. Wiltbank and Pursley [4] achieved contrasting results using a selection of protocols and encouraged simplification. Competitive fertility results (high pregnancy rates and low embryonic loss) were demonstrated in a synchronisation procedure using only two PGF_2α_ analogue treatments, obviating the pre-synchronising steps used to align the follicular waves [5, 6].

Ovarian follicles in cattle are shown to develop in a wave-like pattern, during which the follicles pass through different stages of maturity (emergence, selection, deviation, dominance and atresia or ovulation) over a 7 to-10-day period [7]. Successful synchronisation of ovulation relies on the stage of follicular development at the time of GnRH treatment [8]. In spontaneously ovulating animals, the largest follicle of a follicular wave initiates the process of deviation from other follicles in the same wave approximately 12 h before a difference in their size can be detected [9]. This follicle differs from its contemporaries by having an increase in granulosa LH receptors and oestradiol, rendering it sensitive to the increase in LH following the wave of FSH [9]. In the case of synchronisation of ovulation by GnRH treatment, the diameter of the largest follicle at the time of administration may differ among animals. Interestingly, a positive correlation has been found at the time of GnRH treatment between the size of the dominant follicle, circulating oestradiol and fertility [10]. Correspondingly, animals clearly displaying oestrus were reported to have larger ovulatory follicles [11].

In the past 50 years, the breeding goals concerning Norwegian Red have largely focused on fertility and health in addition milk production, resulting in a relatively high 56 d non-return rate in Norway of approximately 72.5% [12]. Moreover, Norwegian Red has been shown to have an increased oestrus length and more expression of oestrous signs compared to Holstein [13]. An association between the duration of oestrus and ovulatory response has been indicated [14]. Furthermore, cross-bred Holstein and Norwegian Red exhibit high reproductive performance; e.g. enhanced first service pregnancy rates, and considerably fewer days open during their first 5 lactations compared to pure Holstein [15]. Breed differences in oestrous expression and reproductive performance provide an incitement to study the ovulatory response to oestrous synchronisation in Norwegian Red.

The main aim of this study was to examine the ovarian follicular growth, ovulatory response and time to ovulation following synchronisation of oestrus and ovulation in Norwegian Red heifers and cows. Further, effects of herd, age and body condition score (BCS) were examined.

## Methods

### Animals

The experiment was performed in the period from May 2017 to March 2018 in south-eastern Norway. In total, 34 heifers in 4 dairy herds and 10 cows in one of these herds (identified as herd 4), all Norwegian Red cattle, held in free-stalls and fed a mixture of grass silage and grain concentrates supplemented with minerals, were included in this study. The heifers were 14–28 months old and cows were in parities 1–3. At the beginning of the study, the mean (range) for days in milk of the cows was 79.3 (60‒111) and mean daily milk yield (range) was 32.8 kg (23.4‒40.8). Only animals with no record of reproductive, metabolic, mastitis or claw diseases within 2 months prior to the study were included. BCS was recorded and scored on a scale from 1‒5 at the time of the initial treatment of the animals (Additional file 1) by using a visual scoring technique adjusted to Norwegian Red [16]. The study was performed in accordance with the Norwegian Animal Welfare Act (LOV 2009-06-19 no. 97).

### Study design—synchronisation and transrectal ultrasonography

**Fig. 1:**
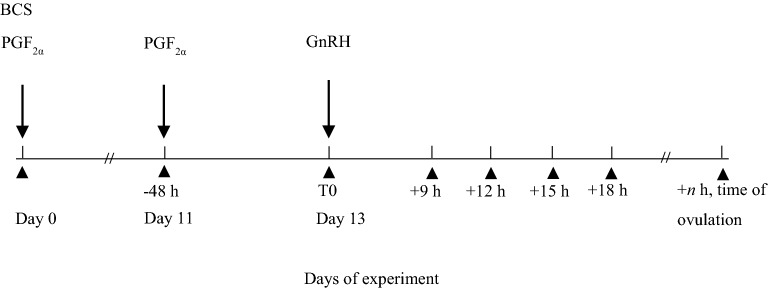
Timeline showing the synchronising protocol and sequence of animal treatments and recordings. PGF_2α_ analogue treatment (PGF_2α_) on day 0 and 11 and body condition scoring (BCS) on day 0; GnRH analogue treatment (GnRH) on day 13 at time 0 (T0); transrectal ovarian ultrasonography (filled triangle) to identify animals with a mature corpus luteum on day 11 (− 48 h) and from day 13 at T0 and every third hour from 9 h following T0 (+ 9 h) until the time of ovulation (+*n* h)

Oestrus cycles of heifers and cows were synchronised with 500 μg cloprostenol (2 mL i.m. Estrumate vet., Intervet International B.V., Boxmeer, the Netherlands), a PGF_2α_ analogue. Estrumate was used for synchronisation according to manufacturer’s instructions and the timing of treatments is illustrated in Fig. 1. Ovarian structures were observed using a BCF Easi-Scan ultrasound scanner fitted with Easi-Scan Smart Display and a 7.5 MHz broadband straight linear rectal probe (BCF Technology Ltd, Scotland, UK). All examinations were conducted by the same veterinarian and assisting colleague. Transrectal ultrasound of the ovaries was used to confirm the presence of a mature corpus luteum (CL) on day 11 of the timeline, identifying animals potentially responsive to the luteolytic effect of the second cloprostenol treatment. One heifer was excluded from the trial at this stage due to lacking a mature CL.

Treatment with buserelin acetate (2.5 mL i.m. Receptal vet., Intervet International B.V., Boxmeer, the Netherlands), a synthetic GnRH analogue, was administered on day 13 at time defined as 0 h (T0). Transrectal ultrasonography of the ovaries was conducted at T0 and every 3rd hour thereafter until ovulation in the first herd (identified as herd 1, n = 9) of the study. In this herd, no animals ovulated within 24 h post treatment with the GnRH analogue. For this reason, ultrasonography following the initial (T0) examination of the remaining herds was postponed 9 h, after which ultrasound examinations every third hour were applied until the animals had ovulated. Following rotation of the rectal probe about the dominant (largest observed) follicle, its position and size [17] was recorded using the electronic callipers of the Easi-Scan Smart Display, until the maximum diameter was found. The persistence of dominant follicles was defined as time elapsed from T0 until their disappearance. Time of ovulation was assumed to be the midpoint between the first ultrasound measurement lacking the dominant follicle and the previous measurement 3 h earlier, i.e. time of the ultrasonography measurement after ovulation minus 1.5 h. Ovulation, i.e. disappearance of the dominant follicle, was confirmed by ultrasound examination and subsequent rectal palpation.

### Progesterone in milk

Milk was sampled from the 10 cows for measurement of P4 [18] in the afternoon (after milking) of day 11 (i.e. time of PGF_2α_ analogue administration) and day 12, and thereafter simultaneously with the ultrasonography examinations. Sampling continued once daily in the afternoon for 2 days following ovulation. All samplings were performed directly after hand strip milking, i.e. milk was drawn from the teat 5 times before sampling. Milk samples (10‒20 mL), preserved with a bronopol tablet, were stored at − 18 °C until analysis. Concentrations of P4 in milk were measured by an enzyme immunoassay [18, 19]. The occurrence of complete luteolysis was detected by measurements showing P4 concentrations < 2.5 ng/mL, as defined earlier [20]. However, one cow had P4 concentrations compatible with incomplete luteolysis, i.e. P4 concentrations remained high (≥ 2.5 ng/mL) within 72 h after PGF_2α_ analogue administration [20] and this sample was therefore removed from the P4 calculations.

### Physical activity of the cows

Activity of the 10 cows was measured individually by an activity monitoring system (DeLaval DelPro, Tumba, Sweden) connected to a central computer in the automatic milking system (VMS, DeLaval). Activity levels were processed by DelPro 5.1 herd management software where activity measured in the past 6 h is compared to activity measured at the same time of day over the past 7 days and expressed as a relative activity ratio. For one of the cows, activity was not recorded due to technical malfunction of the monitoring equipment.

### Data treatment and statistical analyses

Time to ovulation was defined as the number of hours from GnRH analogue treatment to the ultrasonography measurement where ovulation was detected minus 1.5 h. The size of the ovulating follicle was determined from the maximum diameter at the last measurement before ovulation. As ovulations occurred, the number of animals included in calculations for mean (standard deviation) diameter of ovulating follicles were progressively reduced. The data were analysed using Statistical Analysis System (SAS^®^ version 9.4) for Microsoft Windows (SAS Institute Inc., Cary, NC, USA).

#### General linear model analysis

The General Linear Models procedure (SAS^®^ version 9.4) was used to perform a least square analysis for size of the ovulating follicle (outcome variable). The effect of herd, age, BCS and initial diameter of the dominant follicle at T0 on the outcome variable were estimated using the following model:

$$Y_{ijkl} = \mu + H_{i} + A_{j} + B_{k} + I_{l} + e_{ijklm}$$

where *Y*_*ijkl*_ = size of the ovulating follicle in animal *m*; *µ* = overall mean size; *H*_*i*_ = effect of herd, *i* = 1‒4; *A*_*j*_ = effect of age, *j* = 1‒4, where 1 = heifer 14‒15 months, 2 = heifer 16‒18 months, 3 = heifer 19‒28 months, and 4 = cow parity 1‒3; *B*_*k*_ = effect of BCS, *k* = 1‒3, where 1 = (lean: 2.5‒2.9), *k* = 2 (moderate: 3.0‒3.4) or *k* = 3 (fat: 3.5‒4.0); *I*_*l*_ = effect of initial follicle size in 2 classes, where *l* = 1 (diameter ≤ 10 mm) or *l* = 2 (diameter > 10 mm); *e*_*ijklm*_ = residual effect.

A general linear model including the same effects, except that of initial follicle size, and additionally incorporating the size of the dominant follicle at the last measurement was used to analyse time to ovulation (outcome variable).

The study was conducted in herd 1 in May, herd 2 in November, herd 3 in February and herd 4 in February and March. Herd and season could therefore not be separated. Thus, the effect referred to as herd includes possible season effects.

#### Survival analyses

The Lifereg procedure (SAS^®^ version 9.4) was used to perform a Cox regression, thus including time to ovulation for non-ovulating animals (n = 2) as censored animals, rather than missing observations. The possible effects of herd, age, BCS, initial follicle size and size of the ovulating follicle classes as defined above, were tested on the outcome variable (time to ovulation) in the following model:

$$H\left( t \right) = H_{0} \left( t \right) \times \exp \left( {H_{i} + A_{j} + B_{k} + I_{l} + F_{m} } \right)$$

where *H(t)* = the cumulative hazard of ovulating at time *t*; *H*_*0*_*(t)* = the cumulative baseline hazard of ovulating at time *t*; *F*_*m*_ = effect of the size of the ovulatory follicle, *m* = 1‒3, where *m* = 1 (small: 9 < 15 mm), *m* = 2 (medium: 15–17.5 mm) or *m* = 3 (large: 17.5 < 23 mm); *H*_*i*_,* A*_*j*_, *B*_*k*_, and *I*_*l*_ as previously stated.

The hazard ratio is the ratio of the hazard rates corresponding to two levels of one of the predictor variables. By exponentiation of the solution to the base of the natural logarithm (*e*^*b*^), the hazard ratio is found.

## Results

**Fig. 2:**
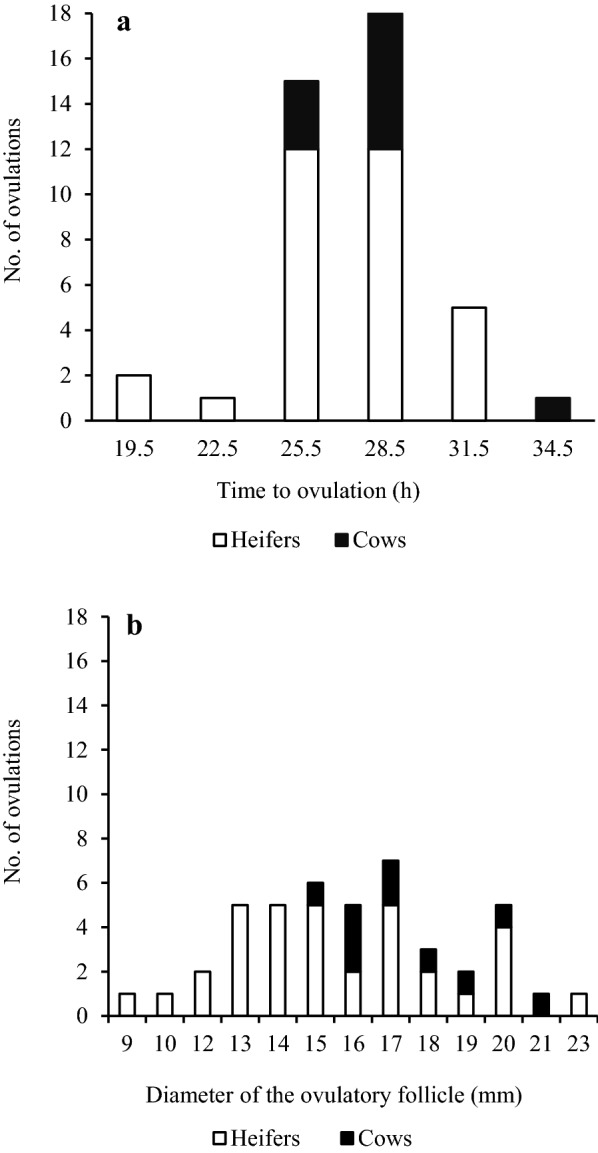
Ovarian follicular response to oestrous synchronisation. Response to PGF_2α_ analogue treatment (twice, 11 days apart) followed by GnRH analogue induction of ovulation 48 h later in heifers and cows. **a** Distribution of time (h) to ovulation measured as time from GnRH analogue treatment to the final ultrasonography examination after ovulation minus 1.5 h and **b** distribution of diameter (mm) of the ovulating follicle (at the last ultrasonography measurement); two heifers did not ovulate

**Fig. 3:**
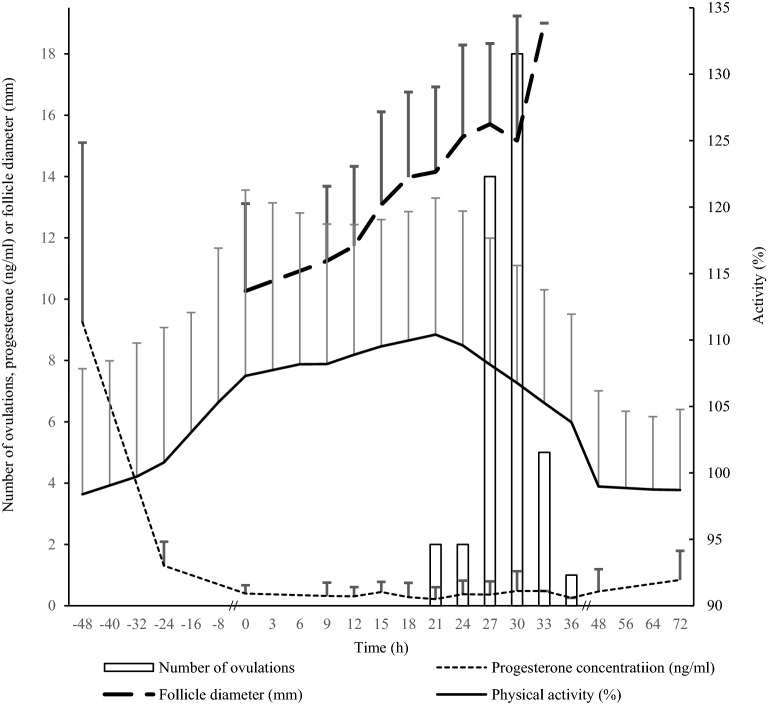
Physical activity, milk progesterone concentrations in cows, and follicular size and time to ovulation in heifers and cows; the results are presented as mean (SD). Mean (SD) in cows (n = 9) for physical activity (%) (activity measured in the past 6 h relative to activity measured at the same time of day in the past 7 days) measured by an automatic activity monitoring system and mean (SD) in progesterone (ng/ml) in milk given by time (h) from the last PGF_2α_ analogue treatment (time = − 48 h); mean (SD) in heifers and cows (n = 32 and 10, respectively) for the diameter (mm) of the dominant follicle measured until ovulation by ultrasonography following GnRH analogue treatment at time = 0 h; number of ovulations at the point of final ultrasonography (1.5 h after ovulation) following GnRH analogue treatment at time = 0 h; two heifers did not ovulate

Of all animals, 95.5% showed an ovulatory response. The mean (SD) time to ovulation for these animals was 27.3 h (3.0), 27.0 (3.1) for heifers and 28.2 (2.6) for cows, with a range of 19.5‒34.5 h (Additional file 2). Distribution of time to ovulation is presented in Fig. 2. The earliest ovulations were observed 19.5 h after T0 (all heifers), whereas most ovulations (72.7% of all animals) were recorded between 25.5 and 28.5 h after T0. The majority of the cows (60%) ovulated 28.5 h after T0. At T0, the mean (SD) diameter of the dominant follicle measured 10.3 mm (2.9). From this point until ovulation, there was a gradual increase in follicle diameter (Fig. 3). The mean (SD) diameter of the dominant follicles measured 1.5 h before the time defined as ovulation was 16 mm (3.0). The diameter at ovulation ranged from 9 to 23 mm (Fig. 2).

The initial mean P4 concentration at − 48 h, was 9.25 ng/mL (range 3.5‒20.4) (Fig. 3). Subsequently, a rapid decline was observed over the following 24 h. Six of 10 cows reached a minimum P4 concentration 66‒69 h after the last PGF_2α_ analogue treatment. From 96 to 120 h after the final PGF_2α_ analogue treatment, P4 concentrations remained low. One cow had particularly high initial P4 concentrations, remaining > 4.0 ng/mL during the whole study (13 samplings from the last PGF_2α_ treatment until 2 days after ovulation), findings which are compatible with incomplete luteolysis [21]. Mean physical relative activity ratios are shown in Fig. 3. The initial measurement at time − 48 h showed a mean activity ratio of 98%, which was the lowest recorded value. Thereafter, the mean activity level increased to 110% 69 h after the final PGF_2α_ analogue treatment, when 5 of 9 cows had reached a maximum activity. A rapid decline in activity ratio was then observed up to 96 h, after which it stabilised.

Effects on the size of the ovulatory follicle are given as least square means (LSM) by herd, age, initial follicle size and BCS in Table 1. Ovulatory follicles were larger in herd 2 than in herd 1 (P < 0.05), and 16 to 18 months old heifers tended to have larger follicles than older heifers (P = 0.06). Large follicles (> 10 mm) at T0 tended to be larger at ovulation compared to other follicles (P = 0.07). The correlation coefficient (95% CI) including the initial size of the dominant follicle and size at ovulation was 0.50 (0.21–0.71). No significant differences were found in LSM for time to ovulation between different levels of herd, age, size of the dominant follicle at the last measurement or BCS.

**Table 1:** Least square means (LSM ± SE) of the size of dominant follicles at ovulation following oestrous synchronisation

Variable	Class	n	Size, LSM ± SE
Herd	1	9	13.2 ± 1.5^a^
	2	10	19.6 ± 1.6^b^
	3	6	14.8 ± 1.5
	4	19	15.8 ± 1.2
Age	Heifers 14‒15 months	10	15.9 ± 1.0
	Heifers 16‒18 months	13	17.1 ± 1.0
	Heifers 19‒28 months	11	13.4 ± 1.5
	Cows parity 1‒3	10	16.9 ± 1.6
Initial follicle size^c^	≤ 10.0 mm	21	15.4 ± 0.7
	> 10.0 mm	23	16.3 ± 0.8
BCS	Lean (2.5‒2.9)	6	15.5 ± 1.7
	Moderate (3.0‒3.4)	20	15.2 ± 0.9
	Fat (3.5‒4.0)	18	16.9 ± 0.8

Number of animals (n) and least square means (LSM) ± standard error (SE) for size of the dominant follicle (diameter, mm) at ovulation by herd, and classes of age, initial follicle size^1^, and body condition score (BCS)

^a,b^LSM within variable and column with different superscripts differ; P < 0.05

^c^Diameter of the dominant follicle at the time of GnRH analogue treatment (T0)

Cox regression analyses showed association between size of the ovulatory follicle and time to ovulation (P < 0.05) (Table 2). Medium-sized and large follicles ovulated earlier than small follicles (P < 0.001 and < 0.05, respectively). Further, heifers in the age groups 16‒18 months and 19‒28 months ovulated earlier than the younger heifers (14‒15 months) (P = 0.05 and P < 0.05, respectively). Animals with a moderate BCS tended to ovulate earlier than fat animals (P < 0.08). In two heifers, no ovulation was observed, although dominant follicles were observed in both animals during the study period. The final observations of these follicles showed that they were surrounded by an approximately 2 mm thin, speckled layer, indicating luteal tissue.

**Table 2:** Survival analysis of time to ovulation following oestrous synchronisation

Variable	Level	Estimate^c^	SE	Hazard ratio (e^*b*^)
Herd	1	–	–	R
	2	0.14	0.12	1.15
	3	− 0.05	0.06	0.95
	4	0.04	0.06	1.04
Age	Heifers 14‒15 months	–	–	R
	Heifers 16‒18 months	− 0.10^a^	0.05	0.90
	Heifers 19‒28 months	− 0.20^a^	0.10	0.82
	Cows parity 1‒3	− 0.02	0.06	1.02
Initial follicle size^d^	≤ 10.0 mm	0.05	0.04	1.05
	> 10.0 mm	–	–	R
Diameter at ovulation^a,e^	9.0‒15.0 mm	–	–	R
	15.1‒17.4 mm	− 0.15^b^	0.05	0.86
	17.5‒23.0 mm	− 0.12^a^	0.06	0.89
BCS	Lean (2.5‒2.9)	0.07	0.07	1.07
	Moderate (3.0‒3.4)	–	–	R
	Fat (3.5‒4.0)	0.08	0.05	1.08

Hazard ratio for time to ovulation by herd, and classes of age, initial follicle size, follicle diameter at ovulation, and body condition score (BCS) in Norwegian Red cattle; time variable: hours from GnRH analogue treatment to ovulation; R, reference value and SE, standard error

^a^Overall association with time to ovulation or within variable difference relative to the reference value; P ≤ 0.05

^b^Within variable difference relative to the reference value; P < 0.001

^c^Estimate for each parameter level

^d^Diameter (mm) of the dominant follicle at the time of GnRH analogue treatment (T0)

^e^Diameter (mm) of the dominant follicle at the time of ovulation

## Discussion

In Norway, until recently, hormone treatment in cattle has been used restrictively, e.g. due to relatively small herds. However, increasing herd sizes makes knowledge on oestrous synchronisation more relevant. In the present study, a gradual increase in mean follicle diameter was recorded from the time of GnRH analogue treatment until ovulation. In total, 95.5% of the Norwegian Red heifers and cows responded to oestrous synchronisation (11 d double PGF_2α_ protocol) and induction of ovulation within 34.5 h of GnRH analogue treatment.

As explained by Meyer et al. [5], two successive PGF_2α_ analogue treatments mediate luteolysis, align the animals into the oestrous cycle, resulting in a marked decline in P4 concentrations, which allows the LH surge that precedes ovulation [22]. In agreement with previous findings [20, 21], 9 of 10 cows in the current study showed a rapid decline in P4 concentrations to values < 2.5 ng/mL within 24 h of PGF_2α_ analogue treatment, indicating complete luteolysis. One cow had milk P4 concentrations indicating incomplete luteolysis yet ovulated within the observed range. This could be due to a postponed but successful luteolysis or variation in P4 concentrations caused by heterogeneity in the time of sampling relative to milking [23]. Considerable individual variations in time to luteolysis after PGF_2α_ treatment have also been reported, likely due to diversity in the stage of oestrous cycles between animals at the time of treatment [24, 25]. Previous research shows an association between failure of luteolysis of seemingly mature CL and high milk yield and low milk protein content [20]. The cows in our study exhibited milk production consistent with earlier findings [20] and above the average in Norway.

Effective oestrous synchronisation relies on ovarian follicular maturity at the time of luteolytic PGF_2α_ treatment relative to the stage of follicular waves and the time of subsequent GnRH induction of ovulation [26]. Onset of ovarian follicular maturity is reported to be observed when the preovulatory follicle reaches about 10 mm in diameter [27]. In the current study, the size of preovulatory follicles at T0 were highly correlated to the size of follicles at ovulation, indicating that ovulatory response to GnRH treatment could be influenced by diversity in the maturity of preovulatory follicles. In the Cox regression, we found that ovulating follicles smaller than 15 mm ovulated significantly later than larger follicles. Our findings on preovulatory follicle size and time to ovulation are in agreement with previous observations [10] showing that small preovulatory follicles were associated with small ovulating follicles and a delayed time of ovulation, possibly caused by physiological immaturity during preovulatory stages. Pre-synchronisation steps using GnRH were not included in the current study, potentially resulting in a greater variability in follicular maturity at the time of GnRH treatment (T0) and subsequent variation in follicular size at ovulation. However, the size of ovulating follicles in our study was comparable to other findings following follicular pre-synchronisation [28, 29], thus questioning the efficiency and necessity of pre-synchronisation steps in healthy animals.

Research in Holstein and Brown Swiss has shown that 80.0% of ovulating cows ovulated within the range of 26‒30 h after GnRH analogue treatment [8]. Correspondingly, a recent study in Holsteins reported that 80.0% of the animals ovulated between 28‒30 h following GnRH analogue treatment [30]. A comparable proportion of animals ovulated within a similar time interval in the current study. Sveberg et al. [13] demonstrated more mounting and standing behaviour during oestrus in Norwegian Red compared to Holstein. Although a reduction in the expression of oestrous activity (e.g. less mounting and standing behaviour) has been observed over the past decades in Holstein compared to Norwegian Red [13], this does not seem to be related to an ovulatory response to synchronisation. Previous findings [28] and results of the present study indicate that the ovulatory response and time to ovulation following oestrous synchronisation is comparable between these dairy breeds. Our results showed a mean time to ovulation from GnRH analogue treatment of 27.3 h, with heifers ovulating slightly earlier than cows. Several factors contribute to the variety in optimal timing of AI, e.g. breed, herd, age and season. In general, AI is recommended within 12–18 h before ovulation [31]. Consequently, our advice is that AI should be performed 9–15 h after induction of ovulation.

## Conclusions

The current study presents new information on the ovarian follicular dynamics and ovulatory response to oestrous synchronisation in Norwegian Red heifers and cows. The applied oestrous synchronisation induced ovulation on average 27.3 h after GnRH treatment in 42 of 44 animals, and 73% of the animals ovulated within a constricted time interval (25‒28 h). In this study the temporal relationships between growth of the ovulatory follicle, time to ovulation, P4 concentrations and physical activity were investigated. Our findings indicate that the response to the applied synchronisation protocol follows a pattern which is in agreement with reports on other breeds. Norwegian Red heifers and cows expressed profiles of ovarian dynamics compatible with successful synchronisation of oestrus and ovulation when GnRH was administered 48 h after administering 2 doses of PGF_2α_ 11 days apart.

## Supplementary information

**Additional file 1.** Body condition score of animals by herd and age. Number of animals (n) mean (SD) and range for body condition score (BCS) grouped by subcategories within the categories herd and age. ^†^BCS was recorded at the initial examination and treatment of the animals by using a visual scoring technique. BCS was measured on a scale from 1 to 5, where 1 is emaciated and 5 is severely over-conditioned animals.

**Additional file 2.** Time to ovulation and size of the ovulatory follicle after oestrous synchronisation. Mean (SD) and range for time to ovulation^†^ and size of the ovulatory follicle^‡^ overall, and in heifers and cows in response to oestrous synchronisation and induction of ovulation^§^; two heifers did not ovulate. ^†^Hours between GnRH analogue treatment at time 0 on day 13 and the first ultrasonography measurement after ovulation minus 1.5 h (midpoint in time between the 3-hourly measurements). ^‡^Diameter (mm) of the dominant follicle at the time of ovulation; two heifers did not ovulate and were excluded from these calculations. ^§^PGF_2α_ analogue treatment on day 0 and 11; GnRH analogue treatment at time 0 on day 13.

## Data Availability

The datasets used and/or analysed during the current study are available from the corresponding author on reasonable request.
