# Supplementary material for: Ovarian follicular response to oestrous synchronisation and induction of ovulation in Norwegian Red cattle
Source: Acta Vet Scand. 2020 Mar 12;62:16. doi: 10.1186/s13028-020-00514-6 (PMC7068941; doi:10.1186/s13028-020-00514-6)
Supplement: Supplementary file 1 — Additional file 1. Body condition score of animals by herd and age. Number of animals (n) mean (SD) and range for body condition score (BCS) grouped by subcategories within the categories herd and age. †BCS was recorded at the initial examination and treatment of the animals by using a visual scoring technique. BCS was measured on a scale from 1 to 5, where 1 is emaciated and 5 is severely over-conditioned animals. [file 13028_2020_514_MOESM1_ESM.docx]

**Additional file 1 Body condition score of animals by herd and age**

| Category | Subcategory | n | BCS^†^(SD) | Range |
| --- | --- | --- | --- | --- |
| Herd | 1 | 9 | 3.6 (0.3) | 3.0–4.0 |
|  | 2 | 10 | 2.8 (0.4) | 2.5–3.5 |
|  | 3 | 6 | 3.4 (0.2) | 3.3–3.8 |
|  | 4 | 19 | 3.3 (0.2) | 3.0–3.8 |
| Age | Heifers, all | 34 | 3.2 (0.4) | 2.5–4.0 |
|  | Heifers 14‒15 months | 10 | 3.2 (0.3) | 2.5–3.5 |
|  | Heifers 16‒18 months | 13 | 3.4 (0.3) | 3.0–4.0 |
|  | Heifers 19‒28 months | 11 | 3.0 (0.5) | 2.5–4.0 |
|  | Cows parity 1‒3 | 10 | 3.5 (0.1) | 3.3–3.8 |
| Total |  | 44 | 3.3 (0.4) | 2.5–4.0 |

Number of animals (N) mean (SD) and range for body condition score (BCS) grouped by subcategories within the categories herd and age

†BCS was recorded at the initial examination and treatment of the animals by using a visual scoring technique. BCS was measured on a scale from 1‒5, where 1 is emaciated and 5 is severely over-conditioned animals.
