# Supplementary material for: Ovarian follicular response to oestrous synchronisation and induction of ovulation in Norwegian Red cattle
Source: Acta Vet Scand. 2020 Mar 12;62:16. doi: 10.1186/s13028-020-00514-6 (PMC7068941; doi:10.1186/s13028-020-00514-6)
Supplement: Supplementary file 2 — Additional file 2. Time to ovulation and size of the ovulatory follicle after oestrous synchronisation. Mean (SD) and range for time to ovulation† and size of the ovulatory follicle‡ overall, and in heifers and cows in response to oestrous synchronisation and induction of ovulation§; two heifers did not ovulate. †Hours between GnRH analogue treatment at time 0 on day 13 and the first ultrasonography measurement after ovulation minus 1.5 h (midpoint in time between the 3-hourly measurements). ‡Diameter (mm) of the dominant follicle at the time of ovulation; two heifers did not ovulate and were excluded from these calculations. §PGF2α analogue treatment on day 0 and 11; GnRH analogue treatment at time 0 on day 13. [file 13028_2020_514_MOESM2_ESM.docx]

**Additional file 2 Time to ovulation and size of the ovulatory follicle after oestrous synchronisation**

| Level | N | Time (SD) | Range | Size (SD) | Range |
| --- | --- | --- | --- | --- | --- |
| Overall | 42 | 27.3 (3.0) | 19.5–34.5 | 16.1 (3.0) | 9.0‒21.0 |
| Heifers | 32 | 27.0 (3.1) | 19.5–31.5 | 15.6 (3.2) | 9.0‒23.0 |
| Cows | 10 | 28.2 (2.6) | 25.5–34.5 | 17.5 (2.0) | 15.0‒21.0 |

Mean (SD) and range for time to ovulation^†^ and size of the ovulatory follicle^‡^ overall, and in heifers and cows in response to oestrous synchronisation and induction of ovulation^§^

^†^Hours between GnRH analogue treatment at time 0 on day 13 and the first ultrasonography measurement after ovulation minus 1.5 h (midpoint in time between the 3-hourly measurements).

^‡^Diameter (mm) of the dominant follicle at the time of ovulation; two heifers did not ovulate and were excluded from these calculations.

^§^PGF_2α_ analogue treatment on day 0 and 11; GnRH analogue treatment at time 0 on day 13.
